# Genitourinary Syndrome of Menopause: Epidemiology, Physiopathology, Clinical Manifestation and Diagnostic

**DOI:** 10.3389/frph.2021.779398

**Published:** 2021-11-15

**Authors:** Ayane Cristine Alves Sarmento, Ana Paula Ferreira Costa, Pedro Vieira-Baptista, Paulo César Giraldo, José Eleutério, Ana Katherine Gonçalves

**Affiliations:** ^1^Postgraduate Program in Health Sciences, Federal University of Rio Grande do Norte, Natal, Brazil; ^2^Lower Genital Tract Unit, Centro Hospitalar de São João, Porto, Portugal; ^3^Hospital Lusíadas Porto, Porto, Portugal; ^4^Department of Obstetrics and Gynecology, State University of Campinas, Campinas, Brazil; ^5^Department of Obstetrics and Gynecology, Federal University of Ceará, Fortaleza, Brazil; ^6^Department of Obstetrics and Gynecology, Federal University of Rio Grande do Norte, Natal, Brazil

**Keywords:** menopause, genitourinary syndrome of menopause, vulvovaginal atrophy, epidemiology, diagnosis

## Abstract

Genitourinary syndrome of menopause (GSM) is a term used to define a compilation of signs and symptoms arising from decreased estrogenic stimulation of the vulvovaginal and lower urinary tract. Among 27–84% of women in postmenopausal are affected for symptoms of GSM, and these can unquestionably impair health, sexual function, consequently the quality of life of these women. The main signs and symptoms of GSM include, among others, burning, irritation, vulvovaginal dryness, dyspareunia, urinary symptoms of urgency, dysuria, or recurrent urinary tract infection. The diagnosis can be made through anamnesis, questionnaires, physical exams, and, sometimes, complementary exams. Objective vaginal assessment is essential and can be complemented by using the Vaginal Health Index (VHI), Vaginal Maturation Index (VMI), or vaginal pH measurement. The acknowledgment of this condition by health professionals is crucial for its identification and proper management and exclusion of other conditions that make a differential diagnosis with it.

## Introduction

Menopause is a condition characterized by estrogen decline and results in numerous changes in the female organism. Vulvar and vaginal atrophy (VA) are among the most common and most bothersome ones (1, 2). The most prevalent symptom is vaginal dryness and affecting 60% of women in the postmenopausal period. In general, these symptoms lead to sexual dysfunction and consequently to reduced quality of life (3, 4).

In 2014, the International Society for the Study of Women's Sexual Health and the North American Menopause Society (NAMS) proposed the introduction of the designation of Genitourinary Syndrome of Menopause (GSM) to replace the term vulvovaginal atrophy (VVA), which has been using for a long time to describe the genitourinary changes of menopause. While several authors and societies use and endorse the concept (5, 6), the justification for its need, its definition, and even its use are still far from consensual. The GSM comprises numerous unspecific symptoms and signs. However, a minimum number of signs/symptoms required for the diagnosis of the syndrome was not defined. One of the major concerns is that given the lack of specificity of the vulvovaginal signs and symptoms, other conditions may easily be overlooked or inappropriate treatments may be provided (7, 8). Another critical question that needs to be taken into account is that VVA can occur also in hypoestrogenic states other than menopause: chemotherapy, pelvic radiation, progestin-only contraceptives, breastfeeding, or anti-estrogenic therapies. In addition, in specific situations, like breastfeeding or certain hormone treatments, it usually is unnecessary to treat women, as the VA resolves spontaneously when estrogen levels are restored (9).

The changes that provoke all the signs and symptoms are correlated with a decline in estrogens and other sex steroids. In general, these changes cause alterations in the clitoris, vaginal vestibule, labia majora and minora, urethra, and bladder. Generally, half of the menopausal and postmenopausal women presenting some signs and symptoms classic of GSM (10). Commonly, the diagnosis is established on the clinical assessment. The latter include anamnesis, assessment of the patient's symptoms, and gynecological examination with an appraisal of clinical signs, including evaluation of sexual function, decreased libido, and dyspareunia. Additionally, standardizer questionnaires and laboratory tests can be employed, such as vaginal pH and the Vaginal Maturation Index (VMI), the latter usually used only in research (11–13).

The increase in life expectancy that has taken place in recent years has meant that female populations live, on average, a third of their life in post-menopause. During this period, symptoms and diseases related to hypoestrogenism become increasingly crucial for women's health. For this reason, good knowledge about the GSM and trusting diagnosis can be valuable tools for the safety and efficacy treatment of these women.

## Search Strategy

We conducted a comprehensive search to the PubMed, Web of Science, Cochrane Library, and Scopus databases using the terms “(menopause OR post-menopause OR genitourinary syndrome of menopause) AND (epidemiology OR physiopathology OR clinical manifestation OR diagnostic OR treatment)” for studies published from inception until July 2021 yielding 5,562 manuscripts. Studies not specific about the themes and other narrative review articles were excluded. Finally, 53 studies were included in the review.

## Epidemiology

Around the world, on average, women live longer than men. Are estimated that these women live more than 30 years following natural menopause, which commonly occurs between 48 and 52 years, in developed countries (14, 15). For this reason, the impact of potential conditions due to menopause-associated hormonal deficiency influences the healthy longevity of women (16, 17). With a prevalence ranging from 36 to 90%, VVA affects many peri- and post-menopausal women. In pre-menopausal years, it can be found in 19% of women aged 40–45 (18–21).

A study involving 2045 British women aged 55–85 years, demonstrated that 50% of these women present urogenital symptoms at some time of menopause. Some of these women were seriously affected; these symptoms are of extended duration and usually do not recede without appropriate treatment (22).

Another pan-European study that enrolled 3,000 women between the ages of 55–75 years and that evaluated the aspects of urogenital aging (UGA) observed that a total of 30% of women experiment from symptoms from urogenital atrophy, these, 60% using hormone therapy (HT) (23). Differences between the six involved European countries were reported, concerning women's perceptions on the use the HT, sexual relationships aspects, prevalence, and treatment of problems due to VVA. Effects of urogenital symptoms on sexual activity were also reported, including a decreased prevalence of sexual activity with increasing age. Over 70% of postmenopausal women were not sexually active, and more than 30% reported dyspareunia and vaginal dryness (23, 24).

Despite the latter result, it is important to refer that in other cohorts it was described that most postmenopausal women continue sexually active. In a study involving 94,000 postmenopausal women (50–79 years), 52% related that who are sexually active with a partner in the last year (25). A review of the literature revealed that 22% of married women between 70 and 79 years still engaged in sexual intercourse (26, 27).

## Physiopathology

The onset of menopause is defined as 12 months without menses. It is a predictable and expected fact in the climacteric. Therefore, endocrine events happen naturally, with its range of symptoms and signs similar to menarche, being also necessary as in this one, a phase of adaptation. There are several changes in ovarian structure and function, with a gradual decrease in estrogen production and a consequent increase in pituitary gonadotropins, characterizing a state of hypogonadotropic hypogonadism (7). During the menopause, the lack of steroid hormones makes the vaginal epithelium thinner due to a decrease in the number of layers of epithelial cells and degeneration of collagen and elastin fibers in the underlying connective tissue, resulting in less tissue elasticity and greater mucosal fragility. The latter changes can provoke burning, fissuring, dyspareunia, and post-coital bleeding. Additionally, the hypoestrogenism induces a decline in the vaginal epithelium glycogen level, which is the substrate for lactobacilli (7, 8).

In women undergoing hysterectomy, the onset of menopause occurs artificially, although the ovaries keep functioning. *In situations* of bilateral oophorectomy, menopause may be accompanied by the clinical manifestations of hypoestrogenism, occurring more frequently and more intensely than in menopause natural (5–8).

## Clinical Manifestations

The vaginal epithelium gets atrophic characteristics in post-menopausal women, presenting surfaces epithelial surfaces with features of keratinization and the absence of rugae, presenting numerous tiers of parabasal cells, and few intermediate and superficial cells (28, 29).

The GSM includes symptoms such as genital alterations of dryness, burning, and irritation, sexual changes, such as the absence of lubrication, discomfort, pain, impaired function until urinary symptoms (urgency, dysuria) (8, 30, 31) (Table 1).

**Table 1:** Clinical manifestations of GSM.

**Genital**	**Sexual**	**Urinary**
**Clinical manifestations**		
Vaginal dryness (21, 24, 32–36)	Sexual dyspareunia (37–40)	Dysuria (41–44)
Irritation/burning/itching (21, 24, 32–36)	Reduced lubrication (37–40)	Urgency (41–44)
Leukorrhea (21, 24, 32–36)	Post-coital bleeding (37–40)	Incontinence (41–44)
Thinning/graying pubic hair (21, 24, 32–36)	Decreased orgasm and desire (37–40)	Stress/urgency (41–44)
Vaginal/pelvic pain and pressure (21, 24, 32–36)	Loss of libido (37–40)	Recurrent urinary tract infections (41–44)
Vaginal vault prolapse (21, 24, 32–36)	Dysorgasmia (37–40)	Urethral prolapse (41–44)
Reduced lubrication (21, 24, 32–36)		Ischemia of vesical trigone (41–44)

*Created by the authors*.

### Genital Symptoms

The vaginal wall presents an abundance of estrogen receptors, especially in the mucosal epithelium, and to a lesser extent in the fibroblasts and the stromal smooth muscle. In the vagina are present two types of estrogen receptors (α and β). The α receptors being present principally in the epithelium, the stroma, and the muscle fibers, while β receptors predominate in the blood vessel endothelium. Besides, androgen receptors also are present in the epithelium and the stroma of the vagina (32–34).

**Figure 1:**
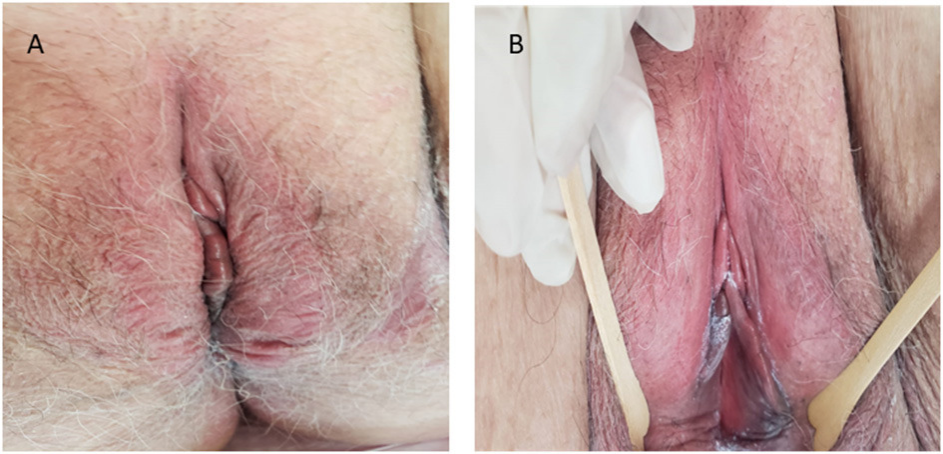
Vulvar during menopause (Authors' personal archive).

Under these circumstances, the vaginal epithelium becomes thinner, inflamed, often with erosions and bleeding (24, 35, 36). Clinical signs associated with histological changes at the vaginal include burning, dryness, loss of elasticity, petechiae, ulceration, inflammation, discharge fibrosis, and vaginal obliteration. The most common vulvar signs include reduction in tissue thickness (loss of fat), labia agglutination, and loss of pubic hair (2, 21, 45) (Figures 1A,B).

### Sexual Symptoms

Coital activity can be uncomfortable in the postmenopausal period because of poor lubrication. In addition, there is a loss of rugae, occasionally stenosis, and the prepuce of the clitoris atrophies (24, 46).

Most common symptoms include vaginal dryness (78%) and superficial dyspareunia (76%). Other symptoms include burning, the pain increased susceptibility to physical and chemical irritation. All of these symptoms affect postmenopausal women's sexuality and relationships (37, 38).

A critical study performed in Spain showed that VVA symptoms significantly affect women's capacity to achieve pleasurable relations (74%) and spontaneity (70%) (39). Another study performed in 2014 demonstrated that most women were concerned discomfort could interfere with your relationship have long-term (40). One crucial point that could aggravate the sexual symptoms is that most women do not seek assistance, considering it's a natural part of aging (24).

### Urinary Symptoms

Due to the bladder trigone and the urethra contain countless estrogen receptors decline in muscle mass and strength when serum estrogen declines provoke the postmenopausal women not to use hormonal therapy to have higher muscle damage (34, 35).

Urogenital symptoms include frequency, nocturia, urgency, and incontinence. Cystitis associated with the estrogen deficit, may not always be acknowledged. The impact of hypoestrogenism is especially relevant in the urethral epithelium, leading to decline sensitivity of urethral smooth muscle, decreased collagen, and loss of urethral wrinkles. The sum of all these changes generally is referred to as the urethral syndrome (41, 42).

Most women (70%) associate the beginning of urinary incontinence with their last menstrual period. Twenty percent refer to grave urgency, and approximately 50% complain of stress incontinence. Urge incontinence is more frequent, and it increases over the years of estrogen deficiency. Shear forces, limited mobility, tissue atrophy, slower dissipation, excess skin hydration, lower tissue regeneration capacity are factors that increase the chances of morbidity development from incontinence dermatitis in postmenopausal women (24, 43, 44).

## Diagnostic

The clinical evaluation of climacteric women should focus on current and past health status and involve a multidisciplinary team. Attention needs encompass health promotion, disease prevention, symptom care clinical and possible difficulties of this phase. Systemic pathologies may occur concomitantly, reverberating in complaints such as joint or muscle pain, gradual weight gain, depression, or even symptoms of undiagnosed hypothyroidism, simulated by coincidence in the ovarian hormonal transition. The VVA diagnosis is based on clinical assessment (anamnesis) and gynecological examination. Besides the use of standardized questionnaires and laboratory exams, such as the vaginal pH and the VMI. Questions about sexual function (including decreased libido), and dyspareunia should be including in the anamnesis (2, 8, 12, 20, 21, 31, 46).

### Vaginal pH

The pH considered normal during the fertile periods of women is among the values of 4.0 and 4.7. The latter occurs due to the presence of lactic acid, which provokes acidifies the medium. In most studies, the vaginal pH, frequently, is measured using a pH indicator strip on the sidewall of the vagina. For this reason, the measurement of vaginal pH is considered useful, effective, and cheap (12, 24, 46–48). However, it is considered unspecific since other factors, such as infections or recent intercourse, can influence the pH vaginal.

### Vaginal Maturation Index (VMI)

The VMI is used to demonstrate the degree of tissue maturation. This measurement is realized for a count of the percentage of superficial, intermediate, and parabasal cells. The vaginal cytology enables a mean index of these three types of cells calculated from at least 100 smear cells. Because the atrophic vaginal tissue can't realize the process of maturation from parabasal and intermediate cells to superficial cells. The percentage of superficial cells correspondents <5%, it indicates an atrophic smear (2, 8, 13, 31, 48, 49). In clinical practice, the use of VMI is unneeded, but it is commonly used in research.

### Vaginal Health Index (VHI)

One of the most common clinical tools used for the evaluation of VVA symptoms is the VHI. This tool evaluates five parameters (vaginal elasticity, vaginal secretions, pH, epithelial mucous membrane, vaginal hydration). The degree of atrophy of the vagina is defined by the final score obtained after the evaluation of each parameter. The final score can vary between 5 and 25, with a cut-off <15 meaning that there is a VA vagina (2, 12, 31, 50).

## Treatment

The main objective of treating GSM is to alleviate symptoms provided by that period. First-line therapies for less severe symptoms include non-hormone vulvar and vaginal lubricants and long-acting vaginal moisturizers used regularly. Although not supported by clinical trials, regular, gentle vaginal stretching exercises may reduce GSM symptoms. Prescription therapies include low-dose vaginal estrogens, vaginal DHEA inserts, and oral ospemifene. For women with moderate to severe dyspareunia associated with GSM with concurrent VMS, transdermal and oral HT are effective options. Pelvic floor muscle training significantly reduces VVA in postmenopausal women. The use of energy-based also has been proposed, and devices are thought to improve vaginal health by causing microtrauma, which induces collagen formation, angiogenesis, and epithelial thickening. The fractional CO_2_ laser has demonstrated safety and efficacy for improving GSM symptoms of vaginal dryness and dyspareunia (6, 8, 32, 49).

Another new proposal includes the use of Visnadine, an active ingredient of the fruit of Ammi visnaga. The latter demonstrates potent vasodilatory activity and has ameliorated female sexual arousal disorder (51). Associated a Visnadine, prenylflavonoids, and phytoestrogens have been considered due to their potential role as estrogen receptor alpha selective agonist and may counteract the effects of postmenopausal estrogen loss. Recently, a vaginal cream containing bovine colostrum has effectively relieved vaginal dryness and other VVA symptoms in postmenopausal women after 8 weeks of treatment (52).

Research realized using the Aloe Vera extract showed a significant improvement in both the VHIS and FSFI, which can be considered direct and indirect signs of increased vaginal epithelialization. All the most significant vaginal health parameters increased indirectly increased female sexual functions too. Finally, the evidence reported that Aloe Vera extracts have substantial antioxidant, antibacterial, anti-inflammatory, and wound-healing properties. Future research is necessary (53).

## Conclusions

GSM is a new term for a condition more renowned as atrophic vaginitis. It is characterized by a hypoestrogenic condition with external genital, urological, and sexual implications that affect postmenopausal women. Knowledge of the concept of GSM and its impact on the quality of life is of paramount importance since it affects millions of women worldwide. The most up-to-date literature about clinical manifestations, pathophysiology, etiology, evaluation and management of GSM is comprehensively reviewed. Early detection and individually tailored pharmacologic or non-pharmacologic. The treatment is paramount for improving quality of life and preventing exacerbation of symptoms in women with this condition. Knowledge of the concept of GSM and its impact on the quality of life is of paramount importance since it affects millions of women worldwide. The GSM is still an under-diagnosed and consequently under-treated condition since the postmenopausal woman does not seek help at the onset of symptoms, use treatments on their own to avoid medical consultation, or do not seek treatment because they believe that all the changes and symptoms are a normal aging process. Due to the latter gaps that still need to be clarified, further and clarifying on this topic are necessary.

## Author Contributions

ACAS and AKG conceived and designed the study. ACAS, AKG, APFC, and PV-B drafted and revised the article where appropriate. ACAS and APFC prepared the Table. AKG, PV-B, JE, and PCG carried out the final revision of the manuscript. All authors contributed to the article and approved the submitted version.

## Conflict of Interest

The authors declare that the research was conducted in the absence of any commercial or financial relationships that could be construed as a potential conflict of interest.

## Publisher's Note

All claims expressed in this article are solely those of the authors and do not necessarily represent those of their affiliated organizations, or those of the publisher, the editors and the reviewers. Any product that may be evaluated in this article, or claim that may be made by its manufacturer, is not guaranteed or endorsed by the publisher.
